# Evaluation of developmental neurotoxicity: some important issues focused on neurobehavioral development

**DOI:** 10.2478/v10102-010-0042-y

**Published:** 2010-11

**Authors:** Michal Dubovický, Pavel Kovačovský, Eduard Ujházy, Jana Navarová, Ingrid Brucknerová, Mojmír Mach

**Affiliations:** 1 Institute of Experimental Pharmacology, Slovak Academy of Sciences, Bratislava, Slovak Republic; 2 Department of Psychology, Philosophical Faculty, Comenius University, Bratislava, Slovak Republic; 3 1^st^ Pediatric Hospital, School of Medicine, Comenius University, Bratislava, Slovak Republic

**Keywords:** neurobehavioral toxicity, neurobehavioral development, behavior, behavioral disorders, open field, habituation, endocrine system, brain, rodents

## Abstract

Exposure of the developing organism to industrial chemicals and physical factors represents a serious risk factor for the development of neurobehavioral disorders, such as attention-deficit hyperactivity disorder, autism and mental retardation. Appropriate animal models are needed to test potentially harmful effects and mechanisms of developmental neurotoxicity of various chemical substances. However, there are significant human vs. rat differences in the brain developmental profile which should be taken into account in neurotoxicity studies. Subtle behavioral alterations are hard to detect by traditional developmental toxicity and teratogenicity studies, and in many cases they remain hidden. They can however be revealed by using special behavioral, endocrine and/or pharmacological challenges, such as repeated behavioral testing, exposure to single stressful stimulus or drugs. Further, current neurobehavioral test protocols recommend to test animals up to their adulthood. However some behavioral alterations, such as anxiety-like behavior or mental deficiency, may become manifest in later periods of development. Our experimental and scientific experiences are highly suggestive for a complex approach in testing potential developmental neurotoxicity. Strong emphasis should be given on repeated behavioral testing of animals up to senescence and on using proper pharmacological and/or stressful challenges.

## Introduction

The central nervous system (CNS), especially the brain, is extremely sensitive during its whole ontogenesis to the action of various chemical substances and/or physical factors. The developing brain has unique characteristics and a different sensitivity to environmental insults compared to the adult already developed brain (O'Rahilly and Müller, 2008). Not only environmental factors but also drugs, stressful stimuli and other factors, such as hypoxia/ischemia, malnutrition, can interfere and/or interact with developmental processes of the brain. These in turn can finally result in subtle structural and/or functional alterations at the level of neurotransmitters and their receptors. Prenatal and perinatal insults, as e.g. developmental exposure to neuroactive drugs and environmental toxins can disturb the timetable of the expression of neurotransmitters and neuromodulators and their receptors. Disruption of the normal timing or intensity of neurotransmitter signaling can lead to permanent changes in proliferation, differentiation and growth of their target cells during the critical phases of brain development, providing potentially the underlying mechanism for neurobehavioral abnormalities (Herlenius and Lagercrantz, 2004). Functional maldevelopment of the brain can manifest as neurological, behavioral, emotional and/or cognitive dysfunctions, which in many cases occur in later development, during childhood, adolescence, adulthood or even in senescence. Attention-deficit hyperactivity disorder (ADHD), mental deficiency, schizophrenia, autism, affective disorders and anxiety were found to be associated with prenatal and early postnatal insults (Casper, 2004; Cannon and Clark, 2005; Langley *et al*., 2005; Kinney *et al*., 2008).

Developmental neurotoxicology deals with the study of adverse effects of chemical substances and physical agents on the developing nervous system. It is important to know the individual stages and factors that are critical in the morphogenetic and functional development of the nervous system, especially the brain, such as neuronal differentiation, migration, cell-cell interactions, neuritic development, synaptogenesis, myelinogenesis, and development of neurotransmitter systems (Slikker and Chang, 1998; Tilson, 2000). One of the marked manifestations of CNS activity is a behavior which is defined as functional adaptation to the environment. Functional alterations of the CNS can occur as behavioral dysfunction in various manifestations. Werboff and Gottlieb (1963) were first to propose the notion of behavioral effects of developmental administration of chemical substances, as well as the term “behavioral teratology”. The authors recognized the importance to use animal studies in evaluation of behaviorally disruptive effects of potential developmental insults (chemicals, drugs, hormones, different environmental impacts). It is actually very difficult to relate a behavioral deficit in children or in adults back to a drug taken by the mothers during pregnancy.

## Developmental neurotoxicity tests

Developmental neurotoxicologists have elaborated several approaches, methods, batteries of special behavioral tests to detect possible behavioral disruptive effects of various factors. There are two basic developmental neurotoxicity (DNT) guidelines: **Health Effects Test Guidelines OPPTS 870.6300 Developmental Neurotoxivity Study** (EPA, 1998) and **OECD Guideline for the Testing of Chemicals, Draft Proposal for a New Guideline 426, Developmental Neurotoxicity Study** (OECD, 1995). DNT studies are based on developmental exposure to a substance tested followed by assessment of growth and developmental landmarks, motor and sensory behaviors, cognitive functions, neurohistopathology, and morphometrics. There are several behavioral test batteries in developmental neurotoxicology. Test batteries are designed mostly for rats. **The Cincinnati Test Battery** is an important screening instrument developed over a period of several years by Vorhees, Butcher and Brunner at the University of Cincinnati (Vorhees *et al*., 1981; 1984). Adams, Buelke-Sam, Kimmel and their colleagues at the National Center for Toxicological research (NCTR) designed a test battery which is based on a large-scale **Collaborative Behavioral Teratology Study** (CBTS) performed by 6 different laboratories around US, including NCTR itself and Vorhees's lab at Cincinnati (Adams *et al*., 1985; Buelke-Sam *et al*., 1985). The CBTS test battery is summarized in Table 1. In Schwerzenbach, Switzerland, J. Elsner, along with his colleagues in Basel and Berlin, Germany, designed a study based on simplified manual tests conducted on young animals and complex automated test procedures performed on adults, known as **European Collaborative Study** (Elsner, 1986; Elsner *et al*., 1986). Fox (1965) performed the first complex investigation of reflex ontogeny and behavioral development in mice (**Fox Battery**). This study served as the basis for much of the subsequent works evaluating neurobehavioral effects of chemical substances in this species. **Most recent behavioral test batteriesin mice** were elaborated by Petruzzi *et al*. (1995) and Rayburn *et al*. (1997), and these include social interaction tests as well.

**Table 1:** The CBTS test battery for rats (Slikker & Chang, 1998)

Measure	Age at measurement (PD)
**Physical growth**	
Body weight	1, 7, 14, 21, 30, 60, 90, 110–120
**Developmental landmarks**	
Upper and lower incisor eruption	7 – completion
Eye opening	12 – completion
Testes descent	21 – completion
Vaginal opening	30 – completion
**Sensorimotor function**	
Negative geotaxis	7–10
Olfactory orientation	9–11
Auditory startle response (habituation)	18–19, 57–58
**Activity and exploration**	
Figure 8 activity (1 hr test)	21, 60
Figure 8 activity (24 hr test)	100–108
**Learning and memory**	
Visual discrimination operant task	75–89
**Pharmacologic challenge**	
Figure 8 activity before and after D-amphetamine	120–131

PD – postnatal day

## Basic procedures of neurobehavioral testing

The main principle of the tests is that the substance tested is administered to females during gestation and lactation and their offspring are randomly selected from litters for neurobehavioral evaluation. At least three dose levels and a concurrent control should be used. The substance tested or vehicle should be administered daily to pregnant females from the time of implantation (day 6 of gestation) throughout lactation up to postnatal day 21, so as to expose the offspring to the substance during their pre- and early postnatal development (OECD, 1995; EPA, 1998). Females are allowed to spontaneously deliver their pups. Before postnatal day 4, the size of each litter should be adjusted by eliminating extra pups by random selection (“culling”) to yield a uniform litter size for all litters, usually 8 pups per litter (4 males and 4 females). Then neurobehavioral development of offspring is evaluated until adulthood, e.g. in the CBTS study, the last variable being tested on postnatal days 120–131. Specialized tests are used for assessment of neurobehavioral development based on ethological analysis of animal behavior. The following categories of neurobehavioral development are investigated: somatic growth and maturation, neuromotor and reflex development, sensory functions, activity and emotionality levels, memory and learning. Individual tests are performed at distinctly established age or time intervals. In some test batteries pharmacological challenge and social behavior evaluation are involved.

**The somatic growth and maturation** category does not represent typical behavioral variables, their observation is nevertheless important to obtain a complex view on developmental processes during early postnatal period. These data are significant for correlation with other developmental landmarks, and in turn for correct interpretation of the results obtained. The following developmental landmarks of maturation are usually observed: body weight, unfolding of external ear, incisor eruption, ear and eye opening, vaginal opening, testes descent.

**Neuromotor and reflex development** tests serve for the evaluation of the level of motor development and its coordination and ability to maintain balance. The variables studied include the crossing of rods of different width, cliff avoidance, the ability so stay on a rotating rod, surface righting, negative geotaxis, dynamic air righting, pivoting and forelimb grip strength.

**Sensory function** tests serve to determine the presence or absence of sensorimotor reactions of the animal, such as olfactory orientation or acoustic startle response.

**Activity and emotional reactivity level** are mostly investigated in anopen field test where intensity of motor activity (ambulation) and rearings is recorded by using various up-to-date techniques, such as video tracking or special infrared beams frames. Time spent in the central and/or peripheral parts of the open field arena and defecation rate are considered the measure of emotional reactivity to stressful stimuli in a new environment. Motor activity should be tested repeatedly to obtain more comprehensive information on animal exploratory behavior in novelty. Repeated testing in open field enables also the evaluation of gradual habituation of animals in a new environment. Consequently, within- and between-sessions habituation processes could be analyzed and compared. Despite the fact that coping behavior in novelty could be a sensitive indicator of behavioral toxicity, complex habituation analyses are omitted in most of the neurotoxicity studies. In our previous studies, we thoroughly investigated habituation of exploratory behavior in neurobehavioral toxicities, and the results obtained are of relevance for evaluating behavioral processes in the open field in a more complex way (Dubovický *et al*., 1997a, b; 1999a, b; 2004).

**Learning and memory processes** testing involves the above mentioned non-associative type of learning, habituation, further classical conditioning, such as active and passive avoidance learning, as well as complex learning processes, such as water maze, radial arm maze learning or visual discrimination operant task.

## Most important issues to be taken into account in neurobehavioral testing

It is important to note that the above mentioned tests are only recommended, they are not mandatory. These tests are not standardized; every neurobehavioral laboratory uses its own test battery, approaching more or less the recommended guidelines of EPA and OECD, or generally used test batteries, such as CBTS. Selection of the tests depends on practical aspects, knowledge, and experience of the investigators, and also on the characteristics of the experiment. In developmental neurotoxicity studies, it is important also to acknowledge the human versus rodent differences, namely differences in developmental processes in the brain, and in social, reproductive and maternal behavior. Due to these differences, chemical substances, drugs and other factors, may interact and/or interfere with developmental processes in the rat brain differently compared to human brain ontogeny. In the following part we mention the most important issues which should be taken into account in developmental neurotoxicity studies.

### Brain growth spurt

Unlike precocial mammals, which give birth to relatively mature and mobile pups, such as guinea pig, hare and most ungulates, the rat, mouse and rabbit belong to the group of altricial species giving birth to immature, immobile, helpless pups. In contrast to precocial species, in altricial mammals the period of intensive brain growth and maturation is shifted to the postnatal period, for rats with the peak on postnatal day 10 – 12. In humans, this brain growth "spurt" peaks in the perinatal period around birth (Dobbing and Sands, 1964; West 1993). The developmental time shift in rats is therefore advantageous for studies of the immature brain. By using neonatal rat pups we can also assess direct effects of various insults on the developing brain, on elimination of the maternal organism. In rats, organogenesis and histogenesis of the brain is completed prenatally and neurogenesis and migration is completed by postnatal day 10. However, synaptogenesis, gliogenesis and myelination, which begin prenatally, continue to develop and they are not completed until maturity (Acuff and Vorhees, 1998). Generally, this fact is not taken into account in developmental neurotoxicity studies. Extension of the dosing period at least to postnatal day 21, when the brain maturation is markedly progressed, postural, locomotor and other skills are completed and pups are able to survive without maternal care and can be weaned (Tilson, 2000), appears to be a strongly substantiated suggestion.

### Neuroendocrine system

The neuroendocrine system enables adaptation of the organism to an ever changing environment. It can be affected during the whole life by various factors. Excessive stressful stimuli can cause neuroendocrine and behavioral alterations in humans as well as in experimental animals (Dubovický *et al*., 1999b; Lanfumey *et al*., 2008). The developing neuroendocrine system is much more sensitive to prenatal and early postnatal insults. Neurotoxicants, intensive stressful stimuli and other factors can affect neuroendocrine and behavioral development in various ways. Alterations in the hypothalamic-pituitary-adrenal axis can be accompanied by long-term neurobehavioral changes. In our previous studies, we found gender-dependent changes in exploratory behavior and habituation in rats neonatally exposed to monosodium glutamate and stressful stimuli (Dubovický *et al*., 1997b; 1999b). Sensitivity of the neuroendocrine system and its investigation from the developmental neurotoxicity point of view is mostly omitted. In many cases neuroendocrine changes could be hidden. Developmental changes can be revealed in reaction of the organism to special stimuli, such as pharmacological challenge or stressful stimuli. In our developmental study with phenytoin, we found an increased responsiveness of the neuroendocrine system in adult rats which were prenatally exposed to this drug. The mild stressful stimulus represented by one-min handling resulted in increased levels of adrenaline and noradrenaline (Makatsori *et al*., 2005). It is important to note that there is evidence that neuroendocrine alterations induced in early development can be associated with depression, anxiety and other stress-related diseases in later life (Maccari *et al*., 2003; Wilcoxon and Redei, 2007). Within neurodevelopmental studies it is relevant to investigate the reactivity of the neuroendocrine system and neurobehavioral variables related to anxiety and depression, such as anxiety- and depression-like behavior of animals in elevated plus maze and test of behavioral despair, respectively.

### Single vs. repeated testing of animals

**Figure 1:**
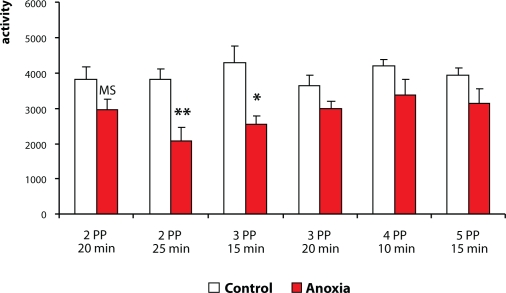
Effect of neonatal anoxia on day 2, 3, 4 or 5 post partum (PP), in duration of 10, 15, 20 or 25 min, on intensity of motor activity of rats in the open field test (single test). MS – marginal significance, *p < 0.05, **p < 0.01 – significant differences compared to controls.

**Figure 2:**
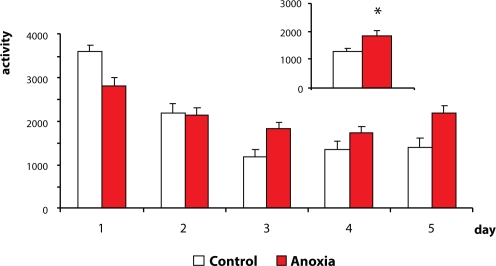
Effect of perinatal asphyxia on day 20 of gestation (non-sophisticated model), lasting 20 min, on intensity of motor activity of rats in the open field test (repeated 5 days of testing). *p < 0.05 – significant differences compared to controls (sum of motor activities on days 3, 4 and 5 of testing).

A test of motor activity is recommended at the time around weaning and repeatedly in adulthood. The tests could be a single one-day session or repeated more-days sessions. As to single or repeated testing, the guidelines are ambiguous. On using these two different approaches, different (sometimes opposite) results can be obtained. In our current experimental practice we test animals repeatedly. Repeated testing in an open field test enables a more complex evaluation of the data obtained, such as habituation processes (Dubovický *et al*., 1997a; 1999a). In a series of experiments in which we investigated the effect of neonatal anoxia on motor activity in the open field at the time around weaning (single anoxia induced on postnatal days 2, 3, 4 or 5 in duration of 10, 15, 20 or 25 min), we found a decrease in motor activity compared to controls in all time intervals. In the study of the effect of perinatal asphyxia on motor activity, we tested weaned rats repeatedly. We found a decrease of the activity on day 1 of testing compared to controls. During the following testing days, however, the activity had a tendency to increase (Dubovický *et al*., 2007). (Figures 1 and 2). These results highly suggest the necessity of repeated testing. A single test is inadequate, since some behavioral processes can be hidden and may become apparent at a later period of life.

### Termination of the neurobehavioral study

Termination of neurobehavioral testing is recommended at the period when animals are sexually mature, in rats it is about postnatal day 60–62. In the CBTS study, figure eight activity before and after D-amphetamine, is scheduled on postnatal days 120–130 (Slikker and Chang, 1998). Prenatal and neonatal insults, however, could manifest themselves in many cases in a later period in adulthood or even senescence. Prenatally administered triethyltin was reported to cause spatial learning impairments at 2 years of age (Barone *et al*., 1995). Some consequences of perinatal asphyxia can appear in old rats, e.g. increased anxiety in senescent rats at the age of 18 months was found by Weitzdoerfer *et al*. (2002). Moreover, there is a relationship between early exposure and increased risk for neurodegenerative and other diseases at higher age (long-term and/or delayed effects). There are neurotoxicity studies confirming the role of developmental neurotoxicant exposure in the induction of neurodegenerative disorders, such as Parkinson's disease (Barlow *et al*., 2007). Other factors, such as stressful stimuli, malnutrition or hypoxia can result in intrauterine growth restriction and in turn can lead to chronic diseases including psychiatric diseases, e.g. schizophrenia. This concept is known as "fetal origin of adult diseases" (FOAD) (Sayer *et al*., 1997; Bezek *et al*., 2008). There is therefore a strong suggestion to shift neurobehavioral testing up to the period of senescence. Although this approach requires life-time testing, the results obtained could yield a better perspective to understand the problem of neurodegenerative and other diseases in maturity and senescence.

## Conclusions

In developmental neurotoxicity studies the different brain developmental profile of rats vs. humans should be taken into account. We would highly suggest to broaden neurotoxicity screening by investigation of the neuroendocrine system subjected to exposure to stressful stimuli in adult life. Evaluation of anxiety- and depression-like behavior appears also to be highly pertinent. In our experience, testing motor activity repeatedly is of utmost importance in order to obtain relevant data on habituation of animals in a new environment. Neurobehavioral tests are not to be terminated in adulthood. They should proceed to senescence so as to reveal potential long-term and/or delayed effects of chemical substances and other adverse factors.

## References

[CIT0001] Accuf KD, Vorhees CV, Niesink RJM, Jaspers RMA, Kornet LMW, van Ree JM, Tilson HA (1998). Neurobehavioral Teratology. Introduction to Neurobehavioral Toxicology: Food and Environment.

[CIT0002] Adams J, Oglesby DM, Ozemek H, Rath J, Kimmel CA, Buelke-Sam J (1985). Collaborative Behavioral teratology Study: programming data entry and automated test system. Neurobehav Toxicol Teratol.

[CIT0003] Barlow BK, Cory-Slechta DA, Richfield EK, Tiruchelvam M (2007). The gestational environment and Parkinson's disease: evidence for neurodevelopmental origins of neurodegenetative disorders. Reprod Toxicol.

[CIT0004] Barone S, Stanton ME, Mundy WR (1995). Neurotoxic effects of neonatal triethyltin (TET) exposure are exacerbated with aging. Neurobiol Aging.

[CIT0005] Bezek S, Mach M, Ujházy E, Dubovický M (2008). Nongenomic memory of foetal history in chronic diseases development. Neuroendocrinol Lett.

[CIT0006] Buelke-Sam J, Kimmel CA, Adams J, Nelson CJ, Vorhees CJ, Wright DC, Omer V, Korol BA, Butcher RE, Geyer MA, Holson JF, Kutscher CL, Wayner MJ (1985). Collaborative Behavioral teratology Study: results. Neurobehav Toxicol Teratol.

[CIT0007] Cannon M, Clarke MC (2005). Risk for schizophrenia – broadening the concepts, pushing back the boundaries. Schizophr Res.

[CIT0008] Casper RC (2004). Nutrients, neurodevelopment and mood. Curr Psychiatry Res.

[CIT0009] Dobbing J, Sands J (1979). Comparative aspects of the brain growth spurt. Early Human Dev.

[CIT0010] Dubovický M, Ježová D (2004). Effect of chronic emotional stress on habituation processes in open field in adult rats. Ann NY Acad Sci.

[CIT0011] Dubovický M, Mach M, Brucknerová I, Ujházy E (2007). Effect of perinatal anoxia on exploratory behavior of rat offspring. Acta Physiol.

[CIT0012] Dubovický M, Škultétyová I, Ježová D (1999b). Neonatal stress alters habituation of exploratory behavior in adult male but not female rats. Pharmacol Biochem Behav.

[CIT0013] Dubovický M, Tokarev D, Škultétyová I, Ježová D (1997b). Changes of exploratory behaviour and its habituation in rats neonatally treated with monosodium glutamate. Pharmacol Biochem Behav.

[CIT0014] Dubovický M, Ujházy E, Kovačovský P, Rychlík I, Kalnovičová T, Navarová J, Turčáni P, Durišová M, Gajdošík A (1997a). Effect of long-term administration of stobadine on exploratory behaviour and on striatal levels of dopamine and serotonin in rats and their offspring. J Appl Toxicol.

[CIT0015] Dubovický M, Ujházy E, Kovačovský P, Rychlík I, Janšák J (1999a). Evaluation of long-term administration of the antioxidant stobadine on exploratory behavior in rats of both genders. J Appl Toxicol.

[CIT0016] Elsner J, Suter KE, Ulbrich B, Schreiner G (1986). Testing strategies in behavioral teratology: IV. Review and general conclusion. Neurobehav Toxicol Teratol.

[CIT0017] Elsner J (1986). Testing strategies in behavioral teratology: III. Microanalysis of behavior. Neuroberhav Toxicol Teratol.

[CIT0018] EPA (1998). Health Effects Test Guidelines, OPPTS 870.6300, Developmental Neurotoxicity Study, EPA 712–C–98–239.

[CIT0019] Fox WM (1965). Reflex ontogeny and behavioral development of the mouse. Anim Behav.

[CIT0020] Herlenius E, Lagercrantz H (2004). Development of neurotransmitter systems during critical period. Exp Nerol.

[CIT0021] Kinney DK, Munir KM, Crowley DJ, Miller AM (2008). Prenatal stress and risk for autism. Neurosci Biobehav Res.

[CIT0022] Lanfumey L, Mongeau R, Cohen-Salmon C, Hamon M (2008). Corticosteroid-serotonin interaction in the neurobiological mechanisms of stress-related disorders. Neurosci Biobehav Rev.

[CIT0023] Langley R, Rice F, van den Bree MB, Thapar A (2005). Maternal smoking during pregnancy as an environmental risk factor for attention deficit hyperactivity disorder behaviour. A review. Minerva Pediatr.

[CIT0024] Maccari S, Darnaudery M, Morley-Fietcher S, Zuena AR, Cinque C, van Reeth O (2003). Prenatal stress and long-term consequences: implications of glucocorticoid hormons. Nerosci Biobehav Res.

[CIT0025] Makatsori A, Dubovický M, Ujházy E, Bakoš J, Ježová D (2005). Neuroendocrine changes in adult female rats prenatally exposed to phenytoin. Neurotoxicol Teratol.

[CIT0026] O'Rahilly R, Müller F (2008). Significant features in the early development of the human brain. Ann Anat.

[CIT0027] OECD (1995). Guideline for the testing of chemicals, Draft proposal for a new guideline 426. Developmental Neurotoxicity Study.

[CIT0028] Petruzzi S, Fiore M, Dell'Omo G, Bignami G, Alleva E (1995). Medium and long-term behavioral effects in mice of gestational exposure to ozone. Neurotoxicol Teratol.

[CIT0029] Rayburn WF, Christenen HD, Gonzales GL (1997). A placebo-controlled comparison between butamethasone and dexamethasone for fetal maturation: differences in neurobehavioral development of mice offspring. Am J Obstet Gynecol.

[CIT0030] Sayer AA, Cooper C, Barker DJ (1997). Is lifespan determined in utero?. Arch Dis Child Fetal Neonatal.

[CIT0031] Slikker W, Chang LW (1998). Hanbook of developmental neurotoxicology.

[CIT0032] Tilson HA (2000). The role of developmental neurotoxicity studies in risk assessment. Toxicol Pathol.

[CIT0033] Vorhees CV, Butcher RV, Brunner RL, Wootten V, Sobotk TJ (1981). Developmental neurobehavioral toxicity of butylated hydroxyanisol (BHA) in rats. Neurobehav Toxicol Teratol.

[CIT0034] Vorhees CV, Butcher RV, Brunner RL, Wootten V (1984). Developmental toxicity and psychotoxicity of sodium nitrite in rats. Chem Toxicol.

[CIT0035] Weitzdoerfer R, Gerstl N, Hoeger H, Mosgoeller W, Dreher W, Engidawork E, Overgaard-Larsen J, Lubec B (2002). Long-term sequele of perinatal asphyxia in aging rats. Cell Mol Life Sci.

[CIT0036] Werboff J, Gottlieb JS (1963). Drugs in pregnancy: behavioral teratology. Obstet Gynecol Survey.

[CIT0037] West JR (1993). Use of pups in a cup model to study brain development. J Nutr.

[CIT0038] Wilcoxon JS, Redei EE (2007). Maternal glucocorticoid deficit affects hypothalamic-pituatury-adrenal function and behavior of rat offspring. Hormon Behav.

